# Potential factors in postoperative dislocation of Oxford phase III mobile bearing UKA in Chinese patients: a single-center retrospective study

**DOI:** 10.1186/s12891-021-04828-y

**Published:** 2021-11-08

**Authors:** Chenkai Li, Tao Li, Zian Zhang, Hui Huang, Tian Chen, Haining Zhang

**Affiliations:** 1 Department of Joint Surgery, The Affiliated Hospital of Qingdao University, Qingdao, 266000 Shandong China; 2 Department of Anesthesiology, The Affiliated Hospital of Qingdao University, Qingdao, 266000 Shandong China; 3 Zhongtai Securities Institute for Financial Studies, Shandong University, Jinan, 250100 China

**Keywords:** Unicompartmental knee arthroplasty, Mobile bearing, Complication, Bearing dislocation

## Abstract

**Background:**

Bearing dislocation is the main complication after mobile bearing unicompartmental knee arthroplasty. The purpose of this study was to analyze the potential risk factors of bearing dislocation after Oxford phase III mobile bearing unicompartmental knee arthroplasty in Chinese patients.

**Methods:**

We retrospectively investigated 492 patients (578 knees) who underwent Oxford phase III mobile bearing unicompartmental knee arthroplasty in our institution between February 2009 and June 2019. The patients were divided into two groups based on surgeons’ annual surgical volume. Those with/ without bearing dislocation were compared based on patient, surgeon and implant factors.

**Results:**

Among the 492 patients, 21 (4.3%, 4 men and 17 women) experienced bearing dislocation. Of these, 14 (4.0%) were in the high surgical volume group and 7 (5.1%) were in the low surgical volume group. Multivariate analysis revealed that trauma to the operated leg and daily life involving high knee flexion cumulatively predicted bearing dislocation (*p* < 0.05).

**Conclusions:**

Trauma to the operated leg and daily life involving high knee flexion were risk factors for bearing dislocation after Oxford phase III mobile bearing unicompartmental knee arthroplasty.

## Background

With the development of minimally invasive surgery (MIS), use of unicompartmental knee arthroplasty (UKA) for isolated medial compartment osteoarthritis (OA) has increased [1]. Compared with total knee arthroplasty (TKA), it has numerous advantages, including better postoperative range of motion (ROM), shorter recovery times, and lower mortality and morbidity [2]. It has been proven that UKA can achieve good clinical outcomes with low postoperative complications.

There are two types of UKA implants, the fixed bearing design and the mobile bearing design. The mobile bearing design, also called the Oxford UKA, was first used for isolated medial compartment OA in 1982 [3]. Compared with the fixed bearing design, the Oxford UKA has a lower wear rate. By using a fully congruent mobile insert, it can reduce contact stresses and transmit compressive forces to the bone-implant interface [4, 5]. Thus, implant wear is decreased and implant lifespan is increased. The wear rates of Oxford UKA have been reported to be 0.06–1.4 mm per year, lower than those of fixed bearings (0.08–1.4 mm per year) [6]. However, bearing dislocation is a postoperative complication that occurs only with Oxford UKA. Furthermore, it has been reported that the incidence of bearing dislocation is higher in Asian populations [2].

This study investigated the risk factors for bearing dislocation after cemented Oxford Phase III UKA performed for medial compartment OA in Chinese patients.

## Methods

### Study setting

Between February 2009 and June 2019, eight surgeons in our single center performed 578 cemented Oxford Phase III UKAs (ZimmerBiomet, Warsaw, IN, USA) for isolated medial compartment OA, in 492 patients. The patients were divided into a high surgical volume group (≥ 15 UKA per year) and a low surgical volume group (<15 UKA per year) based on their surgeons’ annual surgical volume [7]. Patients with/ without bearing dislocation were compared based on patient, surgeon and implant factors. The patient factors were sex, age, body mass index (BMI), lifestyle with/ without high knee flexion (>120°) and history of trauma directly related to the operated leg. Patients’ pre- and postoperative range of motion (ROM) and International Knee Society scores (KSS) were also compared. The surgeon factor was volume of surgery. Implant factors were the thickness of the bearing and implant alignment, which included limb alignment, medial proximal tibial angle (MPTA) and posterior tibial slope (PTS). We obtained approval for this retrospective data analysis from the Medical Ethics Committee of the Affiliated Hospital of Qingdao University.

### Inclusion and exclusion criteria

Patient selection strictly followed the criteria proposed by Goodfellow et al. [8]. Inclusion criteria included a diagnosis of medial compartment OA, failed non-surgical treatment, full-thickness lateral cartilage, intact cruciate ligaments, flexion contracture of less than 15° and fully correctable intra-articular varus deformity. Patients with infection or inflammatory joint disease were excluded.

### Surgical techniques

All surgeons had finished the learning curve and used Oxford Phase III MIS techniques with a medial short incision and limited parapatellar arthrotomy.

### Statistical analyses

SPSS version 24.0 (SPSS Inc., Chicago, IL, USA) was used for statistical analysis. Differences between patients with/without bearing dislocation were analyzed using Student’s t-test; function improvement and clinical outcomes were analyzed using paired Student’s t-tests. Categorical variables were analyzed using the Chi-square test. All analyses were performed using 95% confidence intervals (CIs). Univariate odds ratios were calculated for each of the variables of interest to determine their ability to predict bearing dislocation. Predictive variables that achieved a *p*-value < 0.05 in univariate analysis were entered into backward stepwise multiple regression analysis with revision of the prosthesis as the endpoint of interest. A value of *p* < 0.05 was considered statistically significant.

## Results

Eight surgeons performed 578 UKA in total in 492 patients (125, men, 367, women). Follow-up took place at a mean of 65 months (range, 18-132 months). Of these, 354 patients were in the high surgical volume group and 138 were in the low surgical volume group. None of the patients was lost to follow-up. The demographic characteristics of the patients are shown in Table 1. The daily lives of 140 patients (28.5%) involved high knee flexion, while 15 (3.1%) had a history of trauma directly related to the operated leg. The mean preoperative ROM value was 104.0 ± 10.6°, which improved to 130.1 ± 7.6° by the last follow-up (*p* < 0.0001). The mean preoperative KSS value was 34.8 ± 3.2 and that at the final follow-up was 91.1 ± 4.7 (*p* < 0.0001). The mean thickness of the bearing was 3.96 mm (range, 3 – 8 mm). The mean postoperative mechanical axis was 175.0° (range, 167.7° - 179.9°), and the mean postoperative MPTA and PTS were 86.2° (range, 81.4° - 92.5°) and 9.2° (range, 2.1° - 17.7°) respectively.

**Table 1:** Demographics of patients

Characteristics	
Case number	492 patients (578 UKAs)
Unilateral: bilateral	407: 85
Mean follow-up (months)	65 (range, 18-132)
Gender (male: female)	125: 367
Mean age (years)	61.1 ± 8.4
Mean BMI (kg/m^2^)	27.2 ± 7.8

Regarding the patients without bearing dislocation, their mean age (121 men, 350 women) was 61.2 ± 8.7 years old, and 182 (38.6%) were < 60 years old; mean BMI was 27.2 ± 7.9 kg/m^2^; 205 (43.5%) were obese; the daily lives of 130 (27.6%) involved high knee flexion; and 4 (0.9%) experienced trauma directly related to the operated leg. The mean ROM value improved from 104.0 ± 10.7° preoperatively to 130.1 ± 7.6° at the final follow-up postoperatively (*p* < 0.0001). Mean KSS scores improved from 34.8 ± 3.2 preoperatively to 91.9 ± 4.7 at the latest follow-up (*p* < 0.0001). The mean thickness of the bearing was 3.95 mm (range, 3 – 8 mm). The mean postoperative mechanical axis, MPTA and PTS were 175.1° (range, 167.7° - 179.9°), 86.2° (range, 81.4° - 92.5°) and 9.2° (range, 2.1° - 17.7°) respectively.

**Fig. 1:**
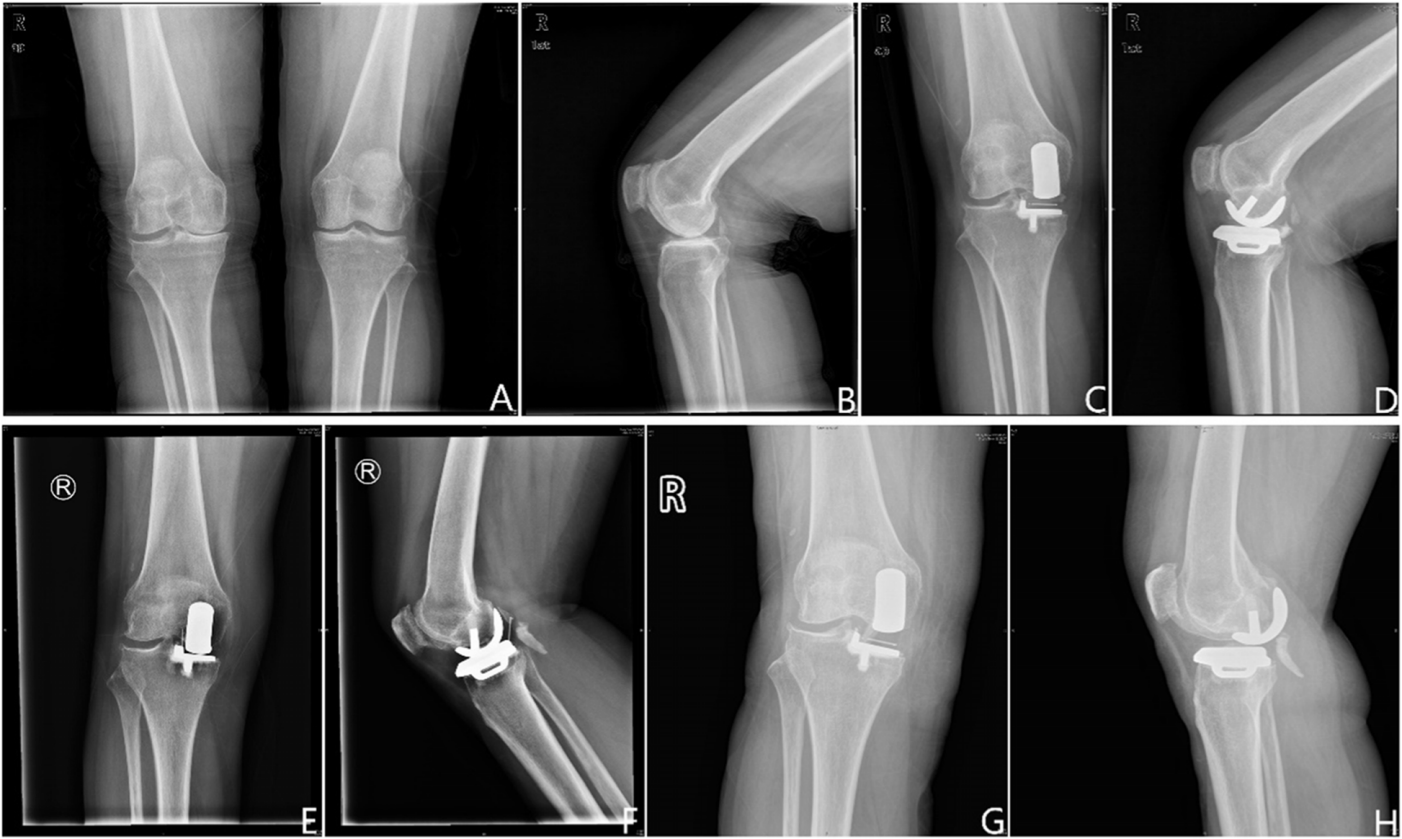
**A**, **B** Preoperative radiographs of the patient. **C**, **D** Postoperative radiographs of the patient after primary UKA. **E**, **F** Posterior bearing dislocation after primary UKA. **G**, **H** Thicker bearing exchange after bearing dislocation

Among the 492 patients, 21 (4.3%, 4 men and 17 women) experienced bearing dislocation (Fig. 1). Of these, 14 (4.0%) were in the high surgical volume group and 7 (5.1%) were in the low surgical volume group. Table 2 shows the demographic characters of the patients who experienced bearing dislocation. Compared with preoperative values, the average KSS score significantly improved from 33.9 ± 2.6 to 92.0 ± 4.4 (*p* < 0.0001), while the men ROM improved from 104.8 ± 10.1° to 131 ± 7.5° (*p* < 0.0001). The mean thickness of bearing was 4.1 mm (rang, 3 – 6 mm). The mean postoperative mechanical axis, MPTA and PTS were 175.1° (range, 167.7° - 179.9°), 85.4° (range, 81.0° - 90.1°) and 9.6° (range, 4.4° - 16.6°) respectively.

**Table 2:** Demographics of patients with bearing dislocation

Characteristics	Bearing dislocation
Case number	21
Unilateral: bilateral	15:6
Gender (male: female)	4: 17
Mean age (years)	59.7 ± 8.0
Mean BMI (kg/m^2^)	26.4 ± 6.5
Direction (anterior: posterior: lateral)	12: 8: 1
Time (after primary operation)	15.9 ± 12.4

We performed a univariate analysis of independent predictors of bearing dislocation and found that trauma associated with the operated leg (*p* < 0.0001) and lifestyle involving high knee flexion (*p* = 0.047) were statistically significant (Table 3). Predictive variables that achieved a *p*-value < 0.10 in the univariate analysis were included in a stepwise backward multiple regression analysis, which showed that trauma associated with the operated leg (*p* < 0.0001) and lifestyle involving high knee flexion (*p* = 0.018) were statistically significant (Table 4). Thus, trauma related to the operated leg and a lifestyle involving high knee flexion cumulatively predicted bearing dislocation. No other predictive variable, achieved statistical significance in univariate or multivariate analysis, or functioned as a confounder or interaction term in the prediction of revision.

**Table 3:** Univariate analysis of predictors of bearing dislocation

Predictors	Odd Ratio	95% CI	*P*-Value
Patient factors			
Gender	1.017	0.363, 2.851	0.974
Age (< 65 years vs ≥65 years)	1.274	0.483, 3.357	0.624
BMI (< 28 kg/m^2^ vs ≥28 kg/m^2^)	2.334	0.841, 6.479	0.095
Life (with high knee flexion vs without high knee flexion)	2.385	0.989, 5.748	0.047
Trauma (surgical leg vs others)	128.425	34.84, 473.398	< 0.0001
Surgeon factor			
High surgical volume vs low surgical volume	0.73	0.287, 1.858	0.508
Implant factor			
Thickness of bearing (Thin-3, 4 mm vs medium- 5,6 mm vs thick- 7, 8 mm)	0.534	0.21, 1.358	0.181
Limb alignment (< 3° vs > 3°)	1.408	0.579, 3.424	0.448
MPTA (84°-90° vs others)	0.649	0.255, 1.654	0.362
PTS (< 7° vs > 7°)	0.922	0.349, 2.434	0.87

**Table 4:** Multivariate analysis of predictors of bearing dislocation

Predictors	Odd Ratio	95% CI	*P*-Value
Life (with high knee flexion vs without high knee flexion)	3.92	1.27, 12.1	0.018
Trauma (surgical leg vs others)	169.455	49.886, 576.616	< 0.0001

## Discussion

UKA for the treatment of anteromedial osteoarthritis (AMOA) has improved since its introduction in the 1950s, and the indications for UKA have continuously expanded due to promising results [9]. However, as the causes of bearing dislocation after Oxford UKA are numerous, multiple factors play significant roles in bearing dislocation. With the expansions of indications, whether the incidence of bearing dislocation has increased remains controversial.

In the past few years, numerous surgeons have expressed concerns about the use of UKA in obese patients, especially the morbidly obese. In 1989, Kozinn et al. [10] recommended a maximum weight of 82 kg for UKA candidates. Deshmukh and Scott suggested 90 kg in 2001 [11]. One study showed that a maximum weight of 80 kg was acceptable, but that clinical outcomes might differ for UKA patients over 90 kg due to increased stress at the implant interface [12]. Polat et al. [5] reported that being morbidly obese was an independent risk factor in UKA. In their study, 10 complications occurred in morbidly obese patients and bearing dislocation occurred in 2 (20%). However, Cepni et al. [13] claimed that a high BMI should not be considered a contraindication and that obese patients could still achieve good clinical outcomes. In our study, there was no significant difference in the incidence of bearing dislocation related to BMI (*p* = 0.095). Although stress increases at the implant interface as body weight increases, a fully congruent bearing decreases the risk of bearing dislocation by transferring pressure to the bone-implant interface. In addition, a mobile bearing also has low contact stress which also decreases the risk of bearing dislocation.

It is generally thought that the UKA revision rate increases with decreasing age, because relatively young patients are more active than older patients. A previous study found that < 60 years of age was a contraindication for UKA [14]; it was also reported that the revision rate after Oxford UKA was 2.9 times greater in patients < 55 years of age compared with those over 75 years of age [15]. However, Kennedy et al. [16] reported that differences between UKA age groups may be associated with the indications for surgery, and that patient age should not be considered a contra-indication for Oxford UKA. Kim et al. [14] reported that it was appropriate for young Asian patients to receive Oxford UKA. In our study, although young patients’ activity levels were higher, the incidence of bearing dislocation was not increased (*p* = 0.624). Both age groups had satisfactory outcomes. In Oxford phase III UKA, both the anterior and posterior rims are elevated, which may restrict excessive movement of the bearing. Therefore, in the absence of impingement or external force, the bearing moves stably.

It has been reported that habitual high flexion postures can result in bearing dislocation [2]. In this case, the lower rim of the medial and lateral parts lose the restriction on motion between the femoral prosthesis and the bearing. Thus, when the knee is suddenly flexed or extended, the residual meniscus, bone cement, or the osteophyte may force the bearing out of its normal position [17]. Kim et al. [18] reported that the risk of bearing dislocation was three times in Asian patients than in Western patients because kneeling or squatting was more common in Asian populations. Multivariate analysis of our follow-up, results showed that high knee flexion was a risk factor for bearing dislocation (*p* = 0.018). When the knee is highly flexed, the stress applied to the bearing and the probability of impingement increase, eventually leading to bearing dislocation. For patients at high risk of bearing dislocation after UKA, surgeons should recommend avoidance of high knee flexion and the use of a knee brace.

Trauma also increases the risk of bearing dislocation (*p* < 0.0001) especially when the ankle is fixed but the knee is twisted. In one study, with a mean follow-up of 12.1 years, UKA required revision in 26 patients, with bearing dislocation being the most common reason (50%) [19]. The injury mechanisms included one in which the lower leg was rotated while sitting straight-legged on the floor and seven that involved standing up after sitting on the floor. The external force acting on the prosthesis or the tissue around the knee joint, reduces the restriction of the bearing and leads to its dislocation. Thus, patients should be advised to avoid trauma, especially sprains, after UKA.

Some recent studies have suggested that early bearing dislocation following UKA is mainly because surgical errors [20, 21]. It was reported that to achieve optimum results, it was appropriate for individual surgeons to perform more than 15 UKA per year or that UKAs should comprise at least 20% (ideally > 30%) of a surgeons’ arthroplasties [7, 22, 23]. In our study, all of the surgeons had finished learning curve and there was no significant difference in bearing dislocation rates between the two groups (*p* = 0.508). However, our results imply that the surgical volume of UKA may be related to clinical outcomes. KSS scores after UKA were 92.1 in the high surgical volume group and 90.6 in the low surgical volume group. While a high surgical volume seemed to be related to better outcomes, the difference was not significant (*p* = 0.2329).

The appropriate size of bearing played an important role in the clinical outcome after UKA. When the bearing is oversized, the medial compartment is overfilled, leading to elevation of the joint line. In contrast, if the bearing is too small, flexion instability occurs which increases the risk of bearing dislocation. In this study, only one patient received a “thick” bearing (8 mm) and he did not experience bearing dislocation. Thus, there was no related statistical analysis. There was no significant difference between “thin” and “medium” bearings (*p* = 0.181). During UKA, the surgeon should select an appropriate bearing that is stable and avoid using a thinner bearing to achieve better knee ROM earlier. In addition, the results show that limb alignment, MPTA and PTS were not risk factors for bearing dislocation (*p* > 0.05). However, implant alignment has been reported to be associated with implant longevity and postoperative complications such as bearing wear, lateral arthrosis, etc. [1, 24].

This study has some limitations. First, this study only included one implant; thus, the results may not be applicable to other implants. Second, the mean follow-up period was 65 months which is relatively short, although most bearing dislocation occurs in the early stage after UKA. Third, all of the surgeons in our study had finished learning curve and the association between trainee and bearing dislocation after UKA remains unknown. Finally, X-ray assessment can be affected by limb rotation, which impacts measurements of implant alignment. Thus, a future study including CT or MRI evaluation is necessary. To elucidate bearing dislocation, long-term or prospective studies are needed.

## Conclusions

The mechanisms of bearing dislocation may not exist in isolation and various factors may work together. To avoid bearing dislocation after Oxford phase III mobile bearing UKA, surgeons should emphasize the risk of bearing dislocation before surgery, and educate patients in detail after operation to avoid trauma and high knee flexion in their daily lives, such as not falling, and wearing kneepads during physical work or exercise.

## Data Availability

The final dataset will be available from the corresponding author.

## Abbreviations

UKA: Unicompartmental knee arthroplasty
TKA: Total knee arthroplasty
ROM: Range of motion
KSS: Knee Society Score
MPTA: Medial proximal tibial angle
PTS: Posterior tibial slope
